# Computer-Assisted Segmentation of Videocapsule Images Using Alpha-Divergence-Based Active Contour in the Framework of Intestinal Pathologies Detection

**DOI:** 10.1155/2014/428583

**Published:** 2014-12-18

**Authors:** L. Meziou, A. Histace, F. Precioso, O. Romain, X. Dray, B. Granado, B. J. Matuszewski

**Affiliations:** ^1^ETIS, Université de Cergy-Pontoise, ENSEA, CNRS, 95014 Cergy-Pontoise Cedex, France; ^2^I3S, Université de Nice/Sophia-Antipolis, CNRS, 06900 Sophia-Antipolis, France; ^3^Paris Sorbonne Paris 7 University & APHP, Hôpital Lariboisière, 75010 Paris, France; ^4^LIP6, Université Pierre et Marie Curie, CNRS, 75252 Paris, France; ^5^Robotics and Computer Vision Research Laboratory, School of Computing Engineering and Physical Sciences, University of Central Lancashire, Preston PR1 2HE, UK

## Abstract

Visualization of the entire length of the gastrointestinal tract through natural orifices is a challenge for endoscopists. Videoendoscopy is currently the “gold standard” technique for diagnosis of different pathologies of the intestinal tract. Wireless capsule endoscopy (WCE) has been developed in the 1990s as an alternative to videoendoscopy to allow direct examination of the gastrointestinal tract without any need for sedation. Nevertheless, the systematic postexamination by the specialist of the 50,000 (for the small bowel) to 150,000 images (for the colon) of a complete acquisition using WCE remains time-consuming and challenging due to the poor quality of WCE images. In this paper, a semiautomatic segmentation for analysis of WCE images is proposed. Based on active contour segmentation, the proposed method introduces alpha-divergences, a flexible statistical similarity measure that gives a real flexibility to different types of gastrointestinal pathologies. Results of segmentation using the proposed approach are shown on different types of real-case examinations, from (multi)polyp(s) segmentation, to radiation enteritis delineation.

## 1. Introduction

Visualization of the entire length of gastrointestinal tract through natural orifices is a challenge for endoscopists. Moreover, radiologic techniques are relatively insensitive for diminutive, flat, vascular, or inflammatory lesions of the small bowel. Currently, videoendoscopy is the “gold standard” technique for the diagnosis of various gastrointestinal diseases. Noticeably, using a videoendoscope, gastroenterologists can perform and record a complete examination of the colon, aiming to detect, to characterize, to sample, and to remove suspicious tissular structures like adenomas (preneoplastic lesions from which colorectal cancer can emerge). In most cases, videocolonoscopy is performed under general anaesthesia. Alternatively, mini-invasive techniques such as computed-tomography-based colonography and wireless capsule endoscopy (WCE) have been proposed when sedation is contraindicated or refused. In this paper, we focus our attention on WCE devices.

**Figure 1:**
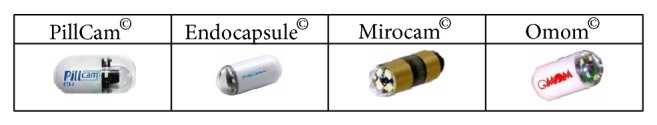
Illustration of the existing WCE devices.

Since 1994 [1], WCEs have been developed to allow direct visualization of the small bowel (a hardly accessible part of the gastrointestinal tract) without any need for sedation. Small-bowel WCEs are best indicated for obscure gastrointestinal bleeding, for evaluation of Crohn's disease and coeliac disease, and when small bowel polyposis (Peutz-Jeghers syndrome) or tumors are suspected. The Pillcam^©^ video capsule designed by Given Imaging Company is the most popular of them (see Figure 1 for illustration).

**Figure 2:**
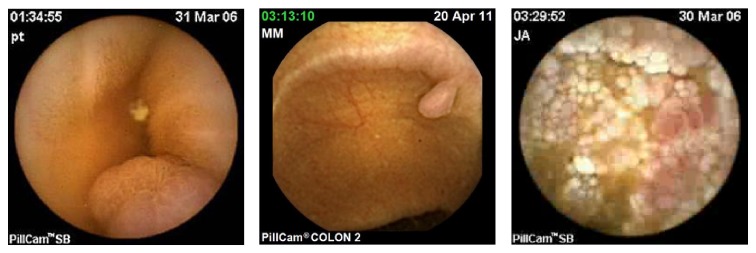
Three images extracted from three different videos made with a Pillcam^©^ WCE (taken from the freely available database “Gastro Vidéo Web” (http://dr.dm.gastro.free.fr/)).

This autonomous device images the gastrointestinal tract during at least 8 hours. However, acquired images are of low resolution and imaging conditions are sometimes very poor, as the movements, the speed of the WCE, and the illumination are not entirely controlled (see Figure 2 for illustration). Moreover a complete acquisition is made of more than 50,000 images for the small bowel (and up to 150,000 for colon WCE), and the analysis of such an amount of data is, therefore, demanding and tedious for physicians. Image segmentation and analysis would be of great interest to decrease the physician's workload.

However, segmentation of WCE images is a challenging problem with two main issues.

1. The substantial reduction in number of images to be analysed is necessary since a lot of them do not show meaningful information from a clinical standpoint and, consequently, do not need to be analysed.
2. The development of an (semi)automatic segmentation approach to robustly and precisely delineate detected structure for further characterization (e.g., size, shape, and texture) is a real need from a computer-assisted diagnostic perspective.

**Figure 3:**
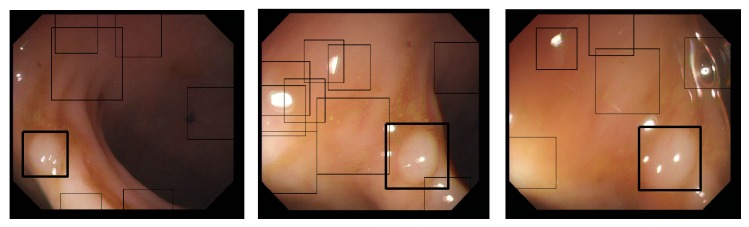
Three examples of the proposed boosting-based polyp detection approach in [2].

Regarding the first issue, in [2], we proposed an automatic boosting-based detection approach for abnormal region of interest (ROI) identification, with a particular focus on polyp detection. A database of 500 polyps and 1200 nonpolyps was used to learn by boosting the optimal classifier in terms of false positive (FP) and true positive (TP) rates. In that study, texture parameters were used (computed from the cooccurrence matrix) and a global detection rate of 94% was reached with a FP rate of 4%. That work, achieved on classic videoendoscopic images, is currently being extended to other types of structure than polyps and to WCE images, for which the image database is far more difficult to build because of limited access to raw images data.  Figure 3 displays three examples of obtained results. In each case, nonbold squares show candidate regions* before* the classifying step, whereas the bold square shows the region eventually identified as a polyp using the boosted classifier. It is important to highlight here that a resolution of only 5 bits (32 grey-levels) was used to compute the cooccurrence matrices from which the Haralick's parameters are then computed. It is shown that, even for low-resolution WCE images (with respect to classic resolution of videocolonoscopy images), the method remains successful.

**Figure 4:**
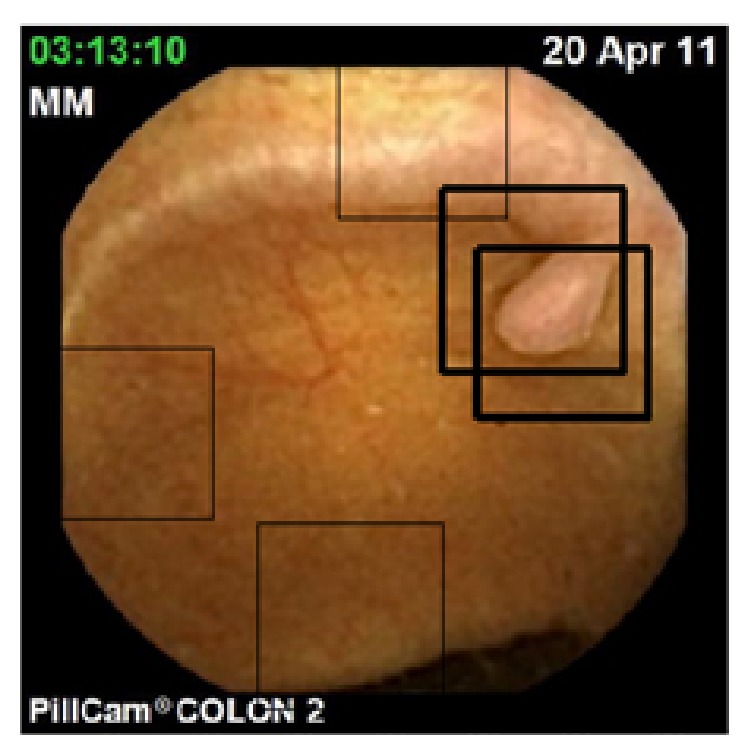
Detection and recognition of a polyp area using the approach proposed in [2] on WCE images. It can be noticed that the only 5-bit resolution of the cooccurrence matrices used for the learning step of the algorithm avoids being too dependent on the “quality” of the image.

To illustrate this, Figure 4 shows a detection/recognition result obtained for one of the WCE images in Figure 2, for which the classifier was trained without using WCE images (currently, most of the WCE manufactures use her proprietary codec for the encryption of the videos, making the collection of images for the database difficult).

Starting from these results, a fine segmentation is required to address the second issue mentioned above. In this paper, a method for WCE image segmentation based on statistical active contours is proposed and a prospective study on various types of gastrointestinal structures is presented.

The remainder of this paper is divided into 4 sections.  Section 2 introduced the data and their specificity in terms of considered pathologies.  Section 3 is focused on the theoretical aspect of the segmentation method proposed. Brief review of histogram-based active contour is first presented and the details on the developed method follow. Segmentation experiments on synthetic images and WCE images are shown in Section 4; the objective of this section is, firstly, to evaluate the method under controlled conditions (noise and texture) and secondly to show comparative performance on the particular targeted clinical application. Finally, conclusion and discussion are proposed in Section 5.

## 2. Materials

Images used for the study purpose were acquired using the Pillcam^©^ device and were taken from the freely available database “Gastro Vidéo Web” (http://dr.dm.gastro.free.fr/). Resolution of images is 320 × 320 pixels.

To show the flexibility of the proposed active contour approach, different segmentation tasks were considered including some demanding ones. Nine conditions were selected for the study (Figure 9): gastric polyps (a, b), colon sigmoid polyp (c), lymphangiectasia (d), small bowel metastasis (e), radiation enteritis (f), angiodysplasia (g), lipoma (h), and hypertrophied anal papilla (i).

In each case, the segmentation task consisted in delineating, with precision and reproducibility, the structure of interest in order to estimate clinical parameters.

## 3. Method

### 3.1. Main Principle and Notations

**Figure 5:**
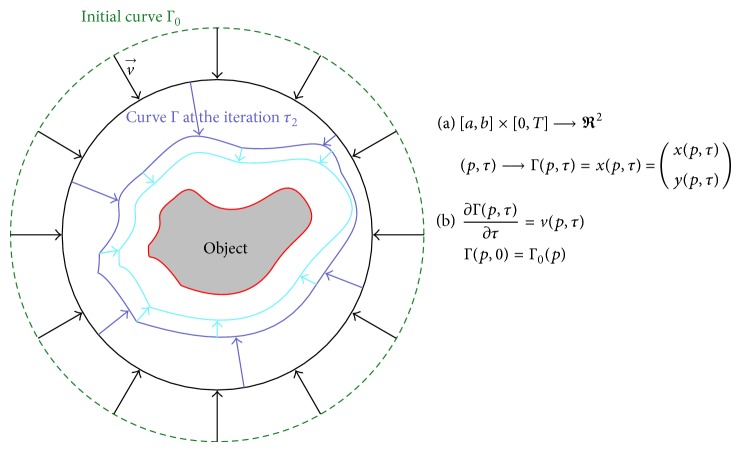
Illustration of active contour segmentation: Γ = Γ(*p*, *τ*) denotes the coordinate of the point *p* of the curve at iteration *τ* of the segmentation process.

Among the existing segmentation methods in medical imaging, active contour models have attracted extensive research in the past two decades. Originally proposed in 1988 [3], the basic idea of the active contour is to iteratively evolve an initial curve towards the boundaries of target organ driven by the combination of internal forces determined by the geometry of the evolving curve and the external forces induced from the image (see Figure 5 for illustration). Image segmentation method using active contour is usually based on minimizing a functional, which is so defined that for curves close to the target boundaries it has small values. The minimization process is iteratively performed until convergence is reached; that is, the value of the computed energy does not meaningfully change between iterations. In the following, we will refer to the active contour and the related evolution equation using the notations (a) and (b) introduced in Figure 5.

Former gradient-based approaches have been now replaced since the early nineties by region-based approaches that are less sensitive to the initialization of the curve, less sensitive to noise, and more stable in terms of obtained results. In that category, the classic Chan and Vese [4] approach is the most used in image segmentation.

**Figure 6:**
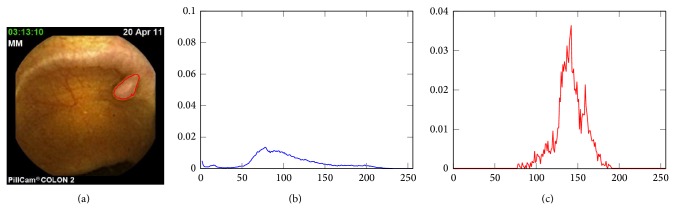
Illustration of the non-Gaussianity of the PDF (luminance) extracted from a WCE image presenting with a polyp. (a) Delineation of the polyp, (b) luminance PDF of the inner (polyp) region, and (c) PDF of the outer (background) region.

Nevertheless, in the particular context of medical image segmentation, the Chan and Vese approach does not suit the particular acquisition noise and more generally the particular imaging conditions; this can be explained by the strong hypothesis of Gaussianity of the luminance distribution law of the inner and outer region of the active curve which is definitely not the case when considering WCE images, for instance. For illustration, Figure 6 shows the luminance distribution function associated to a polyp segmentation task. In blue the inner probability density function (PDF) is shown (*p*_in_) and in red the outer PDF (*p*_out_). As it can be noticed, the PDFs do not adhere with the hypothesis of Gaussianity. In that particular context, histogram-based active contour approaches are of better interest than classic region-based ones.

### 3.2. Alpha-Divergence

In the particular framework of histogram-based active contour segmentation [5–9], external forces involve optimization of distance (or divergence) between the luminance PDF computed from the inner (*p*_in_) and the outer (*p*_out_) regions delimited by the boundaries of the active curve. Commonly the Kullack-Leibler (KL) divergence is used but, as shown in [10], when inner and outer PDFs have too strong overlapping, KL divergence may fail in correctly separating the two regions.

To overcome the limitations of KL divergence, a particular statistical distance is proposed: the alpha- (*α*-) divergence. This similarity measure is a generalization of the KL divergence, which becomes an asymptotic case (*α* tends to one) of the alpha-divergence. Main advantage of proposed approach is the flexibility of the alpha-divergence in which metric can be adapted to very difficult PDF separation tasks [10].

In the following, *D*_*α*_(·||·) will denote the alpha-divergence defined by

(1) $$\begin{array}{c} D_{\alpha }\left(p_{\text{in}}||p_{\text{out}}\right)=\int_{R^{m}}\varphi_{\alpha }\left(p_{\text{in}},p_{\text{out}},\lambda \right)d\lambda \end{array}$$

with *φ*_*α*_ the corresponding alpha-metric such as the following:

(2) φαpin,pout,λ=αpin+1−αpout−pinαpout1−αα1−α,α∈R∖0,1poutlog⁡⁡poutpin+pin−pout,α=0pinlog⁡⁡pinpout+pout−pin,α=1.

In (1), *λ* is the grey-level resolution of the image (i.e., in our case *λ* ∈ [0,255]).

In (1) and (2), it can be noticed that, for particular values of *α*, some usual distances of the literature can be related to alpha-divergences. For instance,

(3) $$\begin{array}{c} D_{2}\left(p_{\text{in}}||p_{\text{out}}\right)=\frac{1}{2}D_{\chi^{2}}\left(p_{\text{in}}||p_{\text{out}}\right)\\ D_{0.5}\left(p_{\text{in}}||p_{\text{out}}\right)=2D_{\text{Hellinger}}\left(p_{\text{in}}||p_{\text{out}}\right)\\ D_{\text{KL}}\left(p_{\text{in}}||p_{\text{out}}\right)=\underset{\alpha \to 1}{\lim}D_{\alpha }\left(p_{\text{in}}||p_{\text{out}}\right), \end{array}$$

where *D*_KL_(·||·) is the KL divergence.

As stated before, this makes alpha-divergence a generic divergence estimation, with multiple tuning possibilities via alpha parameter and, as a consequence, a very flexible measure in the context of non-Gaussian PDF characterizing inner and outer regions delimited by the active curve all along the segmentation process.

### 3.3. PDF Modeling

Practically speaking, the use of alpha-divergence in the framework of histogram-based active contour requires the estimation of the inner and outer PDF. More precisely, the fact that (2) will be optimized using an Euler-derivative approach imposes a continuous and derivable formulation of the PDF. Classically, two kinds of approaches are considered: a parametric estimation or a nonparametric one. In the first case, the PDF can be analytically expressed as part of a general family like the exponential one. Nevertheless, it can be seen as a limitation since, in practical medical case, PDF can be of very complex shapes (e.g., see Figure 6) that cannot be easily expressed in a particular statistical family.

The second solution proposes to estimate the PDF using a Gaussian-mixture model based on Parzen-window estimator [11]. The PDF is then given by

(4) $$\begin{array}{c} p_{i}\left(I\left(x,y\right),\Omega_{i}\right)=\frac{1}{\left|\Omega_{i}\right|}\int_{\Omega_{I}}g_{\sigma }\left(I\left(x,y\right)-\lambda \right)d\lambda \end{array}$$

with *i* ∈ {in, out}, where *Ω*_*i*_ represents all the pixels of region *i* and *g*_*σ*_ represents a Gaussian kernel function of standard deviation *σ*.

Such approach does not limit the type of PDF to be estimated and compelled with the requirement of class *C* ^1^. Such properties definitely fit with our context of applications and in the following Parzen-window technique is used.

### 3.4. Alpha-Divergence-Based Active Contour

As explained above, the segmentation task is formulated as an optimization problem. More precisely, a maximization of (1) using the classic “shape gradients” framework formerly proposed in [5] leads to the following evolution equation, as shown in [12], for nonparametric estimation of the PDF using Parzen-window technique:

(5) $$\begin{array}{c} \frac{\partial \Gamma }{\partial \tau }=\left[\frac{1}{\left|\Omega_{\text{in}}\right|}\left(A_{1}-C_{1}\right)-\frac{1}{\left|\Omega_{\text{out}}\right|}\left(A_{2}-C_{2}\right)\right]N \end{array}$$

with

(6) Aj=∂φαpin,pout,λ∗gσIx,Cj=∫R∂jφαpin,pout,λpidλ,

where {*i*, *j*} = {{in, 1}, {out, 2}}, ∂_1_ *φ*_*α*_ and ∂_2_ *φ*_*α*_ are the derivatives of *φ*_*α*_ with respect to the first (*p*_in_) and the second (*p*_out_) variable, and ∗ denotes the convolution operator.

## 4. Results

### 4.1. Implementation

In order to be able to segment images presenting multiple target objects, we propose to embed the alpha-divergence maximization within the classic level-set framework [13].

Moreover, because a regularization of the obtained curve does not need to take into account small regions in the final segmentation result, a minimization of the final length of the curve is added to (5). Considering the classic level-set embedding function Φ : *R* ^2^ × *R* → *R*, the final evolution equation is then given by

(7) ∂Φ∂τ=δΦ1ΩinA1−C1−1ΩoutA2−C2β·∇·∇Φ∇Φ000000− ξ·1ΩinA1−C1−1ΩoutA2−C2

with *β* and *ξ* the two weighting parameters, *δ*(·) the Dirac function, and ∇ the usual gradient operator. In (7), first term corresponds to the regularization term (minimization of the final length) and second term to the maximization of the alpha-divergence between *p*_in_ and *p*_out_.

For fast implementation under Matlab^©^ 2012, the AOS scheme formerly introduced in [14] was used.

### 4.2. Parameters Setting

As it can be noticed in (7), several parameters, namely, *β*, *ξ*, *σ*, and *α*, influence the optimisation process.

Considering *β* and *ξ*, we arbitrarily set *ξ* to one and then empirically tuned *β* to have a sufficient “amount of energy” for the segmentation process to start and evolve. Main interest of the proposed method is to automatically balance the confidence in the divergence term of (7) using alpha parameter and not being too dependent on *β* value.

*σ* value is empirically tuned to 0.1. A lower value does not bring much to the PDF estimation and a higher value results in a too important smoothing effect on the shape of the PDF (loss of information).

Finally, about alpha parameter that can be seen as the key point of the proposed approach, we propose an automatic setting based on a joint optimization process with (7). More precisely, optimal value of alpha parameter, *α*_opti_, is obtained using a classic gradient descent such as to solve

(8) $$\begin{array}{c} \alpha_{\text{opti}}=\underset{\alpha }{\text{arg max}}\left(D_{\alpha }\left(p_{\text{in}}\right|\left|p_{\text{out}}\right)\right). \end{array}$$

Equations (7) and (8) are optimized in turn. *α*_opti_ value is initialized to 1 in order to start from the classic KL divergence that corresponds to Shannon corresponds to context for which no prior PDF information is taken into account. Main idea is to let the statistical discriminative properties arise from the iterative optimisation process of alpha parameter.

The corresponding proposed algorithm is shown in Algorithm 1.

### 4.3. Experiments

#### 4.3.1. Synthetic Images

**Figure 7:**
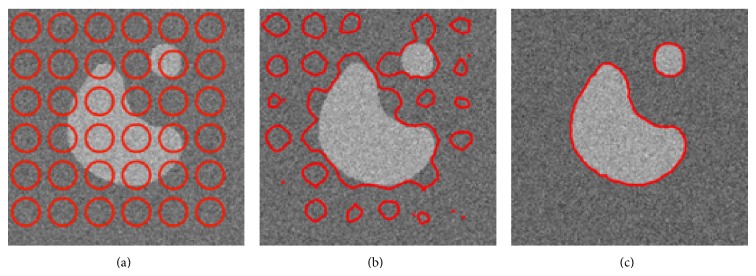
Segmentation results: comparison between KL-divergence-based approach and our method. (a) Original noisy image (Gaussian noise) and initialization of the segmentation process, (b) segmentation result with KL divergence, and (c) segmentation result with proposed approach (*ξ* = 1, *β* = 1, *σ* = 0.1).

First of all, to illustrate the properties of the proposed method, we show the results obtained on a synthetic image (Figure 7). The “peanut” binary shape is corrupted using a strong Gaussian noise such as the PSNR which is only of 10 dB. The segmentation process is initialized using a multiple circles strategy as illustrated in Figure 6(a).  Figure 6(b) shows the segmentation result obtained with the KL divergence and Figure 6(c) results obtained with the proposed approach coupling the maximizations of the alpha-divergence and of alpha value.

As it can be noticed, if KL divergence fails because of the too strong amount of noise, even for Gaussian PDF, proposed method and strategy of optimization for alpha value permit the obtaining of a satisfying segmentation.  Figure 7(b) shows that, with KL divergence, the segmentation process finally stops in a local maximum that does not correspond to the desired segmentation. Optimizing the metric in parallel with the evolution of the active curve makes an adaptation to the statistic properties of the PDF all along the process possible. At convergence, final alpha value is 0.5 in this particular case.

**Figure 8:**
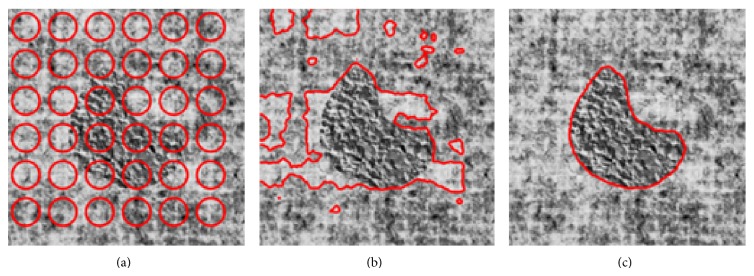
Segmentation results: comparison between KL-divergence-based approach and our method. (a) Original texture image and initialization of the segmentation process, (b) segmentation result with KL divergence, and (c) segmentation result with proposed approach (*ξ* = 1, *β* = 5, *σ* = 0.1).

Figure 8 illustrates performance of the proposed approach on a texture image computed from the Brodatz database. Again, a comparison between KL divergence and our approach is shown.

In that particular case, at convergence optimal alpha value is 0.4. Again, KL divergence fails to segment the peanut shape, being stuck into a local maximum whereas proposed approach leads to the right segmentation.

In terms of robustness, a first statistical evaluation of the final segmentation error (percentage of misclassified pixels) on the peanut shape of Figure 7 was carried out.  Table 1 summarizes obtained results.

Statistics of Table 1 were computed on 100 hundred independent experiments with a different realisation of the Gaussian noise in each case. Proposed method for each experiment outperforms the classic KL divergence and remains stable in terms of final segmentation results.

#### 4.3.2. WCE Image Segmentation

In the following, we now consider application of the proposed approach to the segmentation of different types of structures in WCE images. Considering the initialization issue, we hypothesize that a predetection step permitted automatically identifying the barycentre of particular ROI identified as structure of interest by the learning-based approach of Silva et al. [2] (see Figure 3 for illustration). The initializing curve is a 20-pixel-diameter circle, whose centre is the barycentre of the square in which the structure is included after the detection step.

**Figure 9:**
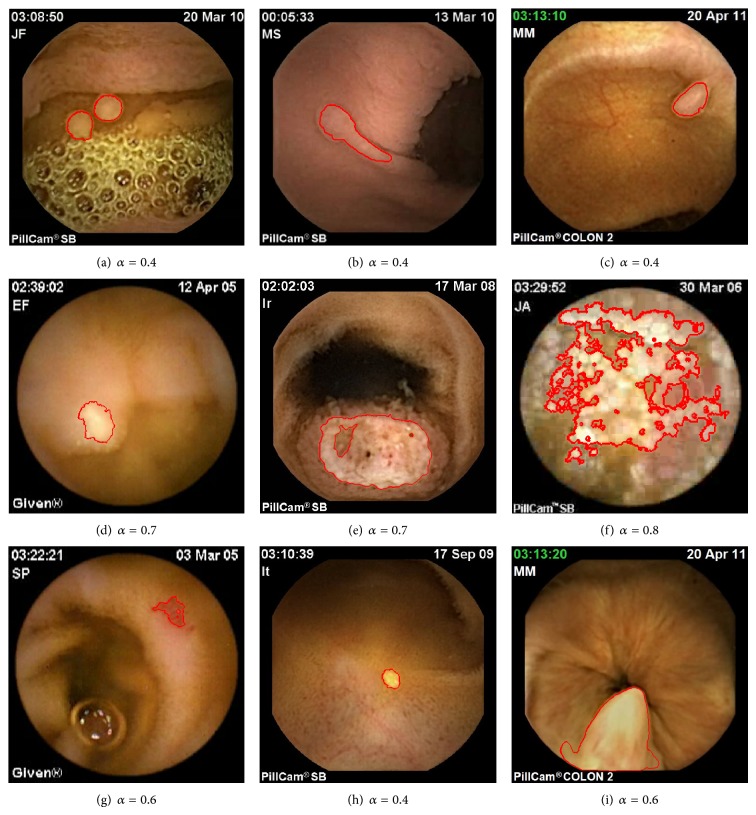
WCE image segmentation using the proposed histogram-based active contour approach. ((a) and (b)) Gastric polyps in Peutz-Jeghers syndrome, (c) colon sigmoid polyp, (d) lymphangiectasia, (e) small-bowel metastasis, (f) radiation enteritis, (g) angiodysplasia, (h) lipoma, and (i) hypertrophied anal papilla. For each segmentation result, indicated values of alpha correspond to the optimal value at convergence of the optimisation process.

For all the experiments, same values of hyper parameters were used (*ξ* = 1, *β* = 10, *σ* = 0.1) except of course for alpha, which was optimized in accordance with the proposed strategy. As it can be noticed, in every case shown, the final segmentation is satisfying and makes the extraction of features possible including, for instance, shape and texture ones. More precisely, as it can be noticed in Figure 9, the maximization of alpha-divergence is a flexible approach that could fit for different kind of segmentation structures acquired in challenging conditions. For instance, even in the case of polyp segmentation, in which luminance characteristics are quite similar to the ones of the associated local background (Figures 9(a)–9(c)), the proposed segmentation approach makes delineation of the boundaries possible whereas usual approach like the Chan and Vese one necessarily fails.

Moreover, as illustrated in Figures 9(d) and 9(f), even if lesions are diffused (like radiation enteritis), the alpha-divergence criterion remains efficient to highlight some particular regions of interest within the considered image. It is also possible to note that the best-obtained results of Figure 9 correspond to value of *α*_opti_ lower than one, that is, different from the asymptotic case *α* = 1 (KL divergence) often used in the particular framework of statistical based active contour.

**Figure 10:**
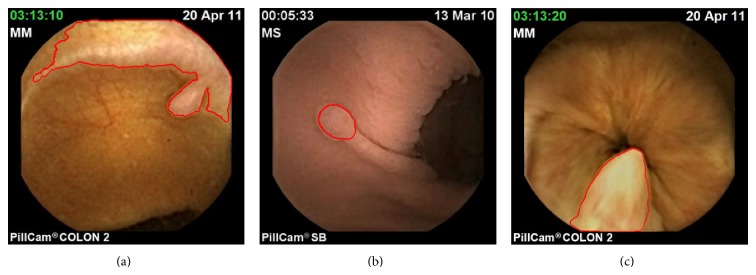
WCE image segmentation using histogram-based active contour approach with KL divergence. (a) Colon sigmoid polyp, (b) gastric polyps in Peutz-Jeghers syndrome, and (c) hypertrophied anal papilla. The same initialisation and hyper parameters were chosen as in the previous experiments.

To allow a visual comparison with KL divergence, we considered some of the most challenging images (mainly polyps) of Figure 9 and performed the segmentation using this similarity measure. Same initialisation and hyper parameters were used. Results are shown in Figure 10.

In each case, it can be noticed that the proposed method outperforms classic KL-based approach.

**Figure 11:**
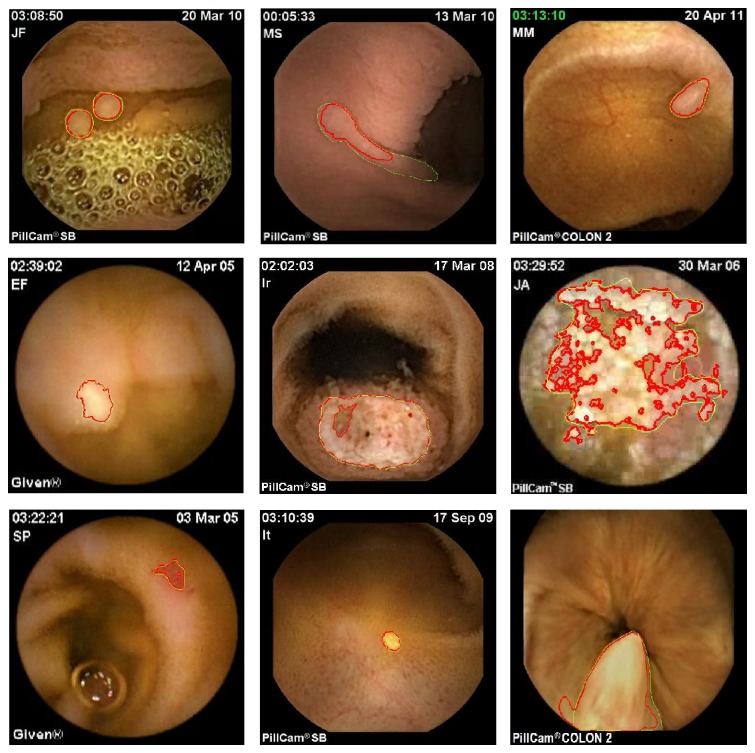
WCE image segmentation of Figure 8 plus, in yellow colour, the manual segmentation performed by the clinician.

Finally, in Figure 11, semiautomatic segmentation results are compared with the corresponding manual delineation performed by the clinicians using the “RatSnake” dedicated free software [15].

We provide here visual results showing that, in most cases, the semiautomatic segmentation is very close to the clinician's one. In addition, in Table 2, for each case, the segmentation error is proposed (computed as the number of misclassified pixels compared to the manual-segmentation mask).

These quantitative results must be considered with care because some values that may be seen as “bad” ones (e.g., with the Gastric polyp (b)) do not highlight the fact that the segmentation obtained with the semiautomatic proposed method is definitely of better interest than the one obtained with KL divergence. One can also notice that, for particular cases like the “enteritis” image or the “metastasis” one, the clinician does not take into account the possible complex topology of the ROI to segment. Because, we use level-set algorithms, the obtained results can highlight particular topological properties that can be of real interest for further characterizations.

## 5. Conclusion and Discussion

In this paper, a flexible statistical region-based active contour criterion has been presented for WCE image segmentation. Based on alpha-divergence similarity, this criterion, embedded into a maximization scheme of the corresponding segmentation energy, shows a real flexibility regarding the particular type of data considered which are acquired in nonusual conditions. The obtained results on a set of WCE images showed that the proposed method overcomes the usual drawback of histogram-based method using KL divergence for similarity measure.

From a clinical point of view, we now need to improve the performance (robustness and reproducibility mainly) on a larger set of data. For this purpose, an acquisition campaign is currently on the run with the clinical partner of the project (APHP, Hôpital Lariboisière, Department of Gastroenterology, Paris Sorbonne Paris 7 University).

Prospective work in terms of image processing will focus on three main issues:

- to take into account colour features of WCE images that can be related to textural information;
- to extract meaningful clinical parameters from the segmented area in order to better differentiate benign from malignant lesions;
- to embed within the capsule the proposed approach, in order to make it compatible with real-time in situ processing.

## Figures and Tables

**Algorithm 1:**
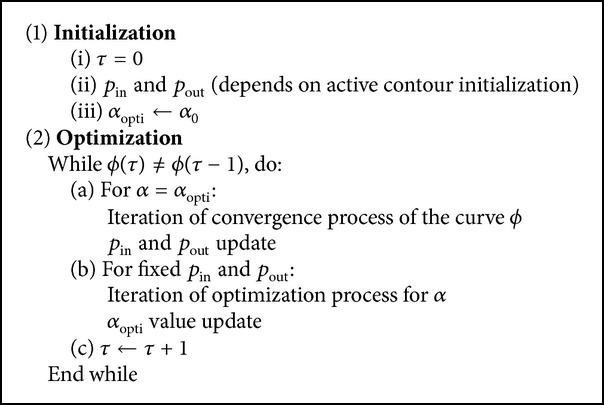


**Table 1:** Average segmentation error in percentage of misclassified pixels function of the strategy used for the setting of *α* parameter (KL or optimized).

Gaussian noise (10 dB)	Segmentation error (%) mean (standard deviation)
KL divergence (*α* = 1)	19.04 (3.4)
*α*_opti_	0.35 (0.02)

**Table 2:** Average segmentation error in percentage of misclassified pixels with respect to the divergence used.

Image	Segmentation error (%)	
	Alpha-divergence	KL
(a) Gastric polyps	<1	
(b) Gastric polyp	44.2	82.3
(c) Colon sigmoid polyp	1.2	~500
(d) Lymphangiectasia	<1	
(e) Metastasis	15.3	
(f) Radiation enteritis	14.7	
(g) Angioma	1.5	
(h) Lipoma	<1	
(i) Anal papilla	11.2	10.3
